# Draft Whole-Genome Sequence and Annotation of the Entomopathogenic Bacterium *Xenorhabdus khoisanae* Strain MCB

**DOI:** 10.1128/genomeA.00872-15

**Published:** 2015-08-06

**Authors:** Stephanie Naidoo, Jonathan Featherston, Vincent M. Gray

**Affiliations:** a School of Molecular and Cell Biology, University of the Witwatersrand, Johannesburg, South Africa; b Biotechnology Platform, Agricultural Research Council, Onderstepoort, South Africa

## Abstract

We report here the draft genome sequence of *Xenorhabdus khoisanae* strain MCB, a Gram-negative bacterium and symbiont of a *Steinernema* entomopathogenic nematode. The genome assembly consists of 266 contigs covering 4.68 Mb. Genome annotation revealed 3,869 protein-coding sequences, with a G+C content of 43.5%.

## GENOME ANNOUNCEMENT

Members of the genus *Xenorhabdus* are motile Gram-negative bacteria belonging to the family *Enterobacteriaceae. Xenorhabdus* species have adopted two distinct lifestyles, one as entomopathogens, and the other as symbionts of entomopathogenic nematodes of the genus *Steinernema* (1). The third infective-stage nematode, the infective juvenile, retains a pellet of *Xenorhabdus* within a specialized intestinal vesicle (2) and in turn serves as a vector in facilitating the transmission of *Xenorhabdus* among susceptible insect hosts (3). Upon invasion by the nematode through natural openings (mouth, anus, or spiracles), *Xenorhabdus* is regurgitated into the hemolymph of an insect, where it rapidly multiplies to high densities (4). The bacteria are able to disable the insect’s defense system (5, 6) and induce death within 48 h through the release of toxins (7, 8). Together, the entomopathogenic nematode and its symbiotic bacteria are of great agricultural importance due to their ability to infect a broad range of soil-inhabiting insects (9). Furthermore, *Xenorhabdus* species serve as good models for understanding parasitism, pathogenicity, and symbiosis. Here, we present a draft genome sequence and annotation of *X. khoisanae* strain MCB associated with the entomopathogenic nematode *Steinernema* sp. HBG28 (GenBank accession no. KJ877686).

*X. khoisanae* MCB was isolated from the hemolymph of *Galleria mellonella* larvae infected with entomopathogenic nematodes, according to the method described by Kaya and Stock (10). Genomic DNA was extracted from solid bacterial colony cultures using a ZR fungal/bacterial DNA MiniPrep kit (Zymo Research). The Nextera DNA sample preparation kit (Illumina) was used in the generation of genomic DNA paired-end libraries. Paired-end (2 × 300 bp) sequencing was performed on an Illumina MiSeq instrument using version 3 MiSeq chemistry at the Agricultural Research Council (ARC) Biotechnology Platform. A total of 2,632,914 paired-end reads were generated from sequencing, which were adapter and quality trimmed using CLC Genomics Workbench version 6.5.1 (CLC bio). The draft genome sequence was assembled *de novo* also with CLC Genomics Workbench version 6.5.1 (CLC bio).

The final assembly consisted of 266 contigs and an *N*_50_ contig size of 38,931 bp. The genome size of *X. khoisanae* was found to be 4,682,720 bp, with a G+C content of 43.5%. The draft genome was annotated using the NCBI Prokaryotic Genome Automatic Annotation Pipeline (PGAAP). The annotation results revealed 4,155 predicted genes and 3,869 protein-coding sequences (CDSs), including 202 pseudogenes, 14 rRNAs, and 65 tRNAs. The genome annotation highlighted the presence of one gene encoding an insecticidal toxin complex protein C, among several other toxin-encoding genes. This protein, in particular, is thought to enhance the cytotoxic effects of another class of insecticidal toxin complexes, referred to as class A proteins (11). We believe that the genome sequence of *X. khoisanae* MCB will result in the discovery of other useful genes and gene products that may be exploited for agricultural application.

### Nucleotide sequence accession numbers.

This whole-genome shotgun project has been deposited at DDBJ/EMBL/GenBank under the accession no. LFCV00000000. The version described in this paper is version LFCV01000000.
